# Resonant inelastic x-ray scattering data for Ruddlesden-Popper and reduced Ruddlesden-Popper nickelates

**DOI:** 10.1038/s41597-023-02079-1

**Published:** 2023-03-29

**Authors:** G. Fabbris, D. Meyers, Y. Shen, V. Bisogni, J. Zhang, J. F. Mitchell, M. R. Norman, S. Johnston, J. Feng, G. S. Chiuzbăian, A. Nicolaou, N. Jaouen, M. P. M. Dean

**Affiliations:** 1 Condensed Matter Physics and Materials Science Department, Brookhaven National Laboratory, Upton, New York, 11973 USA; 2 Advanced Photon Source, Argonne National Laboratory, Lemont, Illinois 60439 USA; 3 Department of Physics, Oklahoma State University, Stillwater, Oklahoma 74078 USA; 4 National Synchrotron Light Source II, Brookhaven National Laboratory, Upton, New York, 11973 USA; 5 Materials Science Division, Argonne National Laboratory, Lemont, Illinois 60439 USA; 6 Institute of Crystal Materials, Shandong University, Jinan, Shandong 250100 China; 7 Department of Physics and Astronomy, The University of Tennessee, Knoxville, Tennessee 37966 USA; 8 Institute of Advanced Materials and Manufacturing, The University of Tennessee, Knoxville, Tennessee 37996 USA; 9 Sorbonne Université, CNRS, Laboratoire de Chimie Physique-Matière et Rayonnement, 75005 Paris, France; 10 Synchrotron SOLEIL, L’Orme des Merisiers, Saint-Aubin, BP 48, 91192 Gif-sur-Yvette, France; 11 Present Address: Institute of Advanced Science Facilities, Shenzhen, Guangdong 518107 China

**Keywords:** Electronic properties and materials, Superconducting properties and materials

## Abstract

Ruddlesden-Popper and reduced Ruddlesden-Popper nickelates are intriguing candidates for mimicking the properties of high-temperature superconducting cuprates. The degree of similarity between these nickelates and cuprates has been the subject of considerable debate. Resonant inelastic x-ray scattering (RIXS) has played an important role in exploring their electronic and magnetic excitations, but these efforts have been stymied by inconsistencies between different samples and the lack of publicly available data for detailed comparison. To address this issue, we present open RIXS data on La_4_Ni_3_O_10_ and La_4_Ni_3_O_8_.

## Background & Summary

**Fig. 1:**
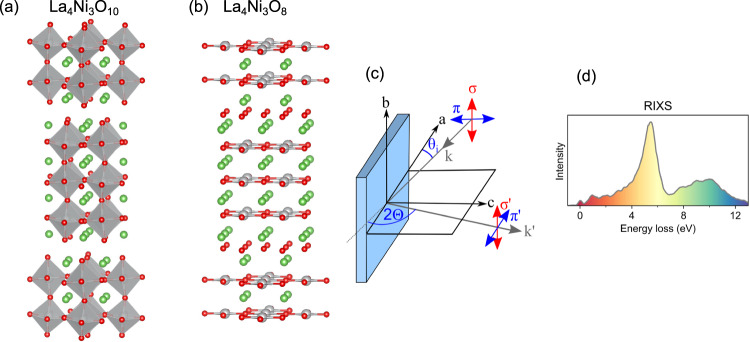
Summary of study. (**a**,**b**) Crystal structure of (**a**) La_4_Ni_3_O_10_ and (**b**) La_4_Ni_3_O_8_. La, Ni, and O atoms are depicted by green, gray, and red spheres, respectively. (**c**) The experimental geometry showing the incident x-ray angle *θ*_i_, the scattering angle 2Θ, and incident x-ray polarization *π* and *σ*. (**d**) An example RIXS spectrum, which encodes the electronic structure of these materials.

Generating analogs of the copper-based high-temperature superconductors based on different transition metal ions has been a major goal of materials research since the 1980s, as this opens routes to improving our understanding of unconventional superconductivity and realizing materials with improved properties^1–3^. Since nickel lies immediately to the left of copper in the periodic table, it represents an obvious target element for realizing new superconductors. However, despite extensive work, no superconductivity has been found in nominally Ni 3*d*^8^ or 3*d*^7^ compounds^4–6^. One route to realizing the desired 3*d*^9^ state found in copper-based superconductors in a nickel oxide has been achieved by preparing members of the Ruddlesden-Popper phase materials with formula *R*_*n* + 1_Ni_*n*_O_3*n* + 1_ (where *R* is a rare earth element) and reducing them through removal of their apical oxygen atoms, forming *R*_*n* + 1_Ni_*n*_O_2*n* + 2_ materials [see Fig. 1a,b]. Indeed, this approach has been validated by the discovery of superconductivity in infinite layer *n* = ∞ *R*_1-*x*_Sr_*x*_NiO_2_, and subsequently *n* = 5 Nd_6_Ni_5_O_12_, generating intense scientific interest^7–10^.

Resonant x-ray spectroscopy offers several advantages for probing low valence nickelates^11^. It can probe small sample volumes, which is useful since these materials are challenging to prepare as large single crystals. Indeed, many low valence nickelate samples are only available in thin film form. Another unique feature is the ability of RIXS to selectively study different atomic species through the resonant process and its access to magnetic and orbital excitations, which are optically forbidden^12^. Efforts to understand the detailed electronic properties of Ruddlesden-Popper and reduced Ruddlesden-Popper nickelates have consequently relied heavily on resonant x-ray spectroscopy^11,13–23^.

However, much of this debate has been complicated by two major difficulties. The first is inconsistent spectra from nominally equivalent or very similar samples, an issue raised specifically in refs. ^18,24^. The second is the lack of open data, which precludes detailed direct comparison between samples. Herein, we provide RIXS data from bulk crystals of La_4_Ni_3_O_10_ (*n* = 3) and La_4_Ni_3_O_8_ (*n* = 3, after reduction). In addition, little publicly accessible advice is currently available for comparing RIXS datasets, so we take this opportunity to outline considerations in comparing RIXS data taken under different experimental conditions.

## Methods

The parent Ruddlesden-Popper La_4_Ni_3_O_10_ was synthesized using the high pressure optical floating zone method at Argonne National Laboratory. During growth, 0.1 l/min of oxygen gas was flowed through the reaction chamber at 20 bar pressure and the feed rods were advanced at 4 mm/h over the 30 hour growth time. To improve homogeneity, the feed and seed rods were counter-rotated at 30 r.p.m. throughout this process. A similar procedure was used in ref. ^25^. The *c*-axis surface used in the measurement was mirror polished in water using diamond lapping paper. La_4_Ni_3_O_8_ single crystals were prepared by cleaving small crystals from the parent La_4_Ni_3_O_10_ crystals and reducing them by heating in a flowing 4% H_2_/Ar gas mixture at 350°C for five days. This procedure was used successfully in several prior works^14,16,20,22,25^.

Ni *L*_3,2_ RIXS as well as O *K* and Ni *L*_3,2_ x-ray absorption spectroscopy (XAS) measurements were performed at the AERHA instrument of the SEXTANTS beamline at the SOLEIL synchrotron. The spectrometer operates by dispersing the x-ray photons as a function of their energy onto a two-dimensional detector via the detailed optical scheme described in ref. ^26^. A sketch of the experimental geometry is displayed in Fig. 1c. All measurements were performed with the *a* and *c* sample axes in the horizontal scattering plane. At the Ni *L* edges, data were taken at *θ*_*i*_ = 15° degrees (*θ*_*i*_ is the angle between the incoming x-ray and the sample surface), the linear dichroism is obtained by switching the incident x-ray polarization between *π* and *σ*. For the O *K* edge, a fixed *π* x-ray polarization was used, the dichroism is obtained by measuring at *θ*_*i*_ = 15° and 90°. RIXS was measured at 2Θ = 95°, with an overall full-width at half-maximum (FWHM) energy resolution of 262 ± 9 meV, chosen in order to balance throughput and resolution. As is standard in modern RIXS experiments, the two-dimensional data are binned in the isoenergetic direction to form spectra and the pixel to energy loss conversion is performed by measuring the elastic line of the spectrometer while changing the beamline energy. The data describing this calibration are provided in the Technical Validation section.

## Data Records

**Fig. 2:**
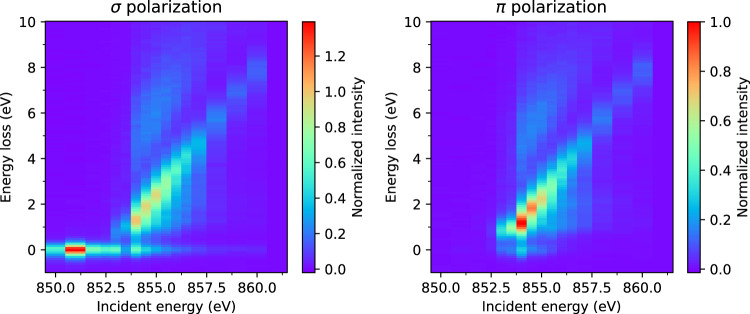
Ni *L*_3_-edge RIXS energy map for La_4_Ni_3_O_10_ measured at 25 K with an incident angle of *θ*_*i*_ = 15° and scattering angle of 2Θ = 95°. The left and right panels show *σ* and *π* incident x-ray polarization, respectively.

**Fig. 3:**
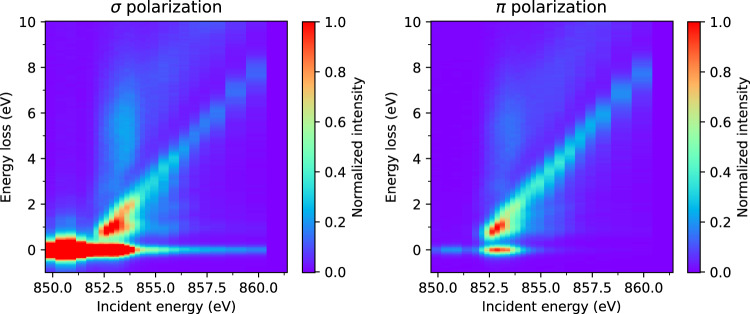
Ni *L*_3_-edge RIXS energy map for La_4_Ni_3_O_8_ measured at 25 K with an incident angle of *θ*_*i*_ = 15° and scattering angle of 2Θ = 95°. The left and right panels show *σ* and *π* incident x-ray polarization, respectively.

**Fig. 4:**
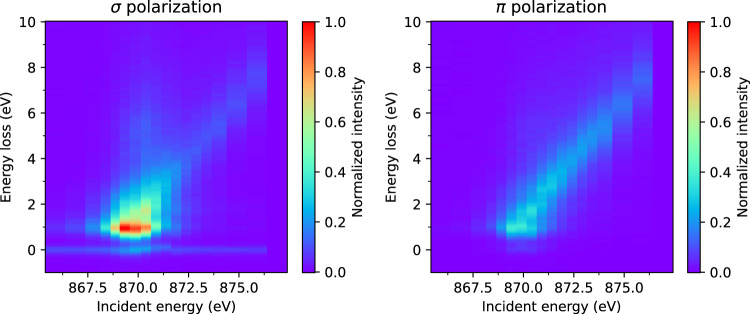
Ni *L*_2_-edge RIXS energy map for La_4_Ni_3_O_8_ measured at 25 K with an incident angle of *θ*_*i*_ = 15° and scattering angle of 2Θ = 95°. The left and right panels show *σ* and *π* incident x-ray polarization, respectively.

**Fig. 5:**
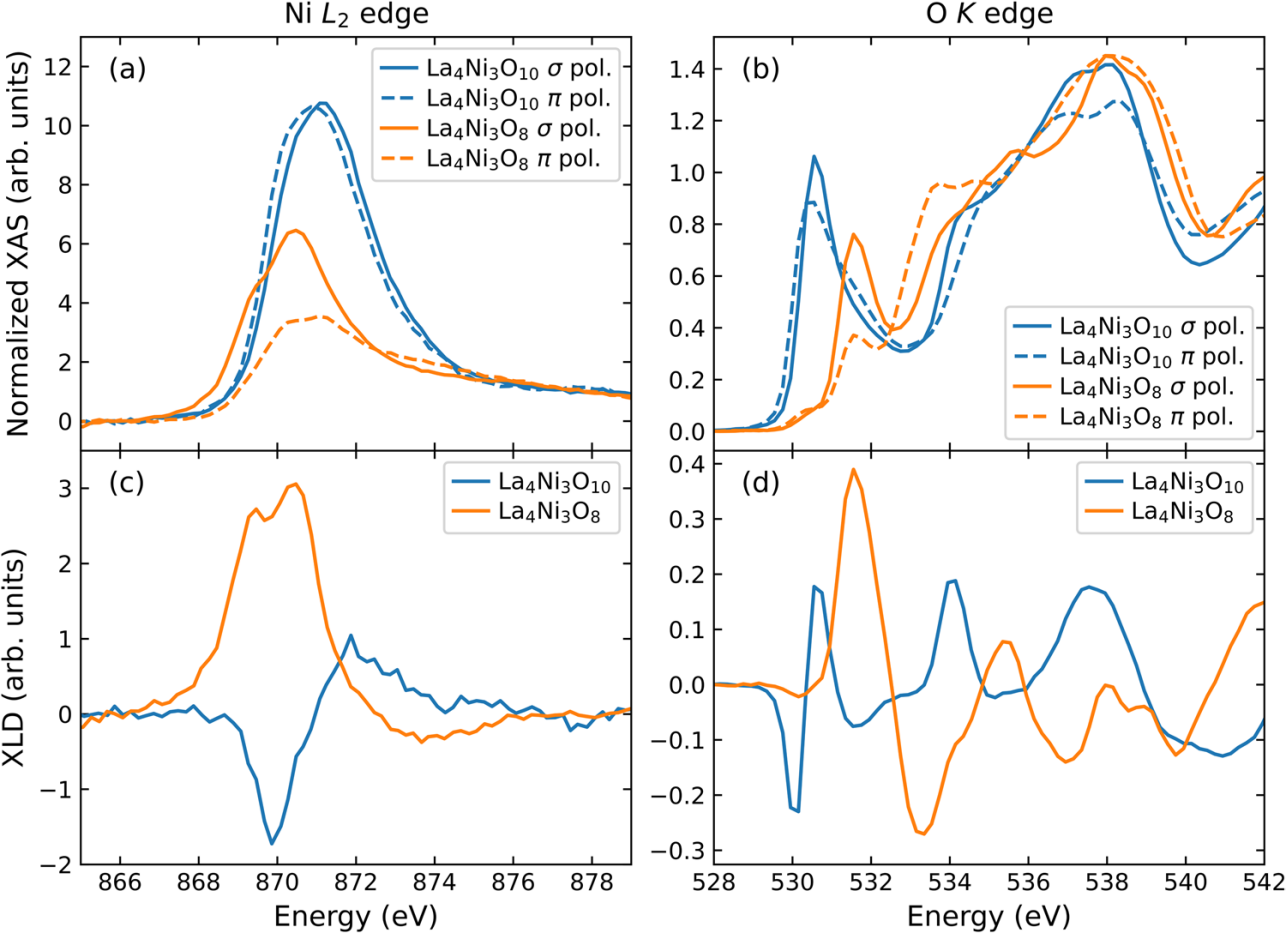
XAS measurements of La_4_Ni_3_O_10_ and La_4_Ni_3_O_8_ taken at 300 K and *θ*_*i*_ = 15°. The Ni *L*_2_ edge XAS and XLD are displayed in panels (**a**,**c**), respectively. Panels (**b**,**d**) display the O *K* edge XAS and XLD, respectively.

**Fig. 6:**
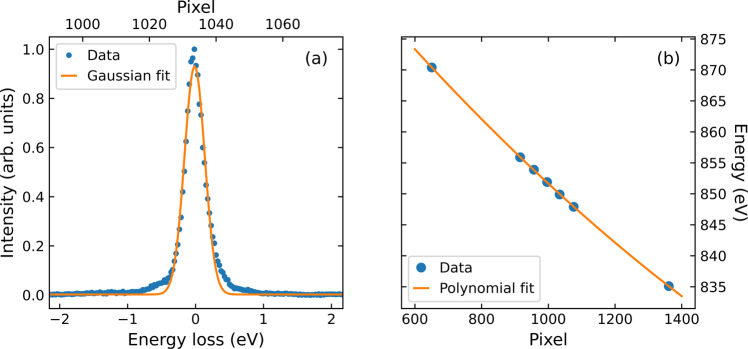
RIXS instrument parameters. (**a**) Elastic line measurement; a gaussian fit yields FWHM = 262 ± 9 meV. (**b**) Dispersion of the elastic line through the CCD detector, the calibration follows a second order polynomial (*y* = *a* + *bx* + *cx*^2^) with *a* = 912.7 eV, *b* = −7.24 × 10^−2^ eV/pixel, and *c* = 1.13 × 10^−5^ eV/pixel^2^.

This Data Descriptor is based on data deposited in the Zenodo general-purpose open repository^27^. RIXS data are provided as text files with filename of the form La4Ni3O10_pi_850.0eV.dat, which specify the material, incident x-ray polarization, and incident energy. This information as well as the sample temperature are provided in the file header. XAS data are also provided as text files with a similar nomenclature, but which includes the absorption edge measured, for instance La4Ni3O8_sigma_OKedgeXAS.dat. These data are also plotted in Figs. 2–4. The XAS data displayed in Fig. 5 were taken in total fluorescence yield (TFY), but files also contain data taken in total electron yield (TEY) concomitantly. Finally, the data shown in Fig. 6a,b are stored in files elastic_850eV.dat and pixel_energy_dispersion.dat, respectively. The repository illustrates the data content using plotting scripts based on standard python stack of numpy, matplotlib, scipy, and pandas^28–30^. The jupyter-repo2docker is used to define the computational environment and making the code executable on services such as mybinder.org.

## Technical Validation

Figure 5 displays the Ni *L*_2_ and O *K* edge XAS and x-ray linear dichroism (XLD) of La_4_Ni_3_O_10_ and La_4_Ni_3_O_8_. The larger XAS white line area seen in La_4_Ni_3_O_10_ and the larger XLD in La_4_Ni_3_O_8_ are consistent with previous results^14^. A similarly large XLD in La_4_Ni_3_O_8_ is observed at the O *K*-edge [Fig. 5d]. These results confirm the quality of the samples used here. The energy resolution of the RIXS measurement was determined by fitting a Gaussian peak to a spectrum collected at an energy away from the resonance [Fig. 6a]. The data collected by the CCD area detector was converted to energy space using the calibration shown in Fig. 6b. Since the La *M*_4_-and Ni *L*_3_-edges overlap in energy, we also provide data at the Ni *L*_2_-edge in Fig. 4.

## Usage Notes

Several technical issues make it quite difficult to measure RIXS in absolute units. We therefore present data in arbitrary units. We suggest comparing the preset spectra to other works after dividing both spectra by the integrated spectral intensity in the 0.5–15 eV energy range. It is better to exclude the elastic line from such comparisons, as the quasielastic intensity is particularly sensitive to extrinsic issues such as sample surface flatness.

To compare the data presented here with other data, care should be taken to account for possible differences in energy resolution between the different datasets. We suggest comparing data by convolving the higher-resolution spectrum with a lineshape that gives both spectra the same effective resolution.

## Data Availability

The data reported here were generated via synchrotron experiments and did not require any processing of datasets beyond trivial binning of the two-dimensional data into one-dimensional spectra and calibration of the energy loss.
